# The Role and Therapeutic Targeting of JAK/STAT Signaling in Glioblastoma

**DOI:** 10.3390/cancers13030437

**Published:** 2021-01-24

**Authors:** Alexander Ou, Martina Ott, Dexing Fang, Amy B. Heimberger

**Affiliations:** 1 Department of Neuro-Oncology, University of Texas M.D. Anderson Cancer Center, 1515 Holcombe Blvd., Houston, TX 77030, USA; AOu@mdanderson.org; 2 Department of Neurosurgery, University of Texas M.D. Anderson Cancer Center, 1515 Holcombe Blvd., Houston, TX 77030, USA; MOtt@mdanderson.org (M.O.); DFang@mdanderson.org (D.F.)

**Keywords:** JAK, STAT3, glioblastoma, immunotherapy, resistance

## Abstract

**Simple Summary:**

Glioblastoma is one of the most treatment-refractory human malignancies, and despite techniques that have allowed scientists and clinicians to better understand the molecular underpinnings of resistance, little progress has been made in improving the survival of patients with glioblastoma. We posit that dysregulated Janus kinase/signal transducer and activator of transcription (JAK/STAT) signaling represents one major hub of tumorigenesis and resistance to medical therapies and that clinical study of its targeted inhibition is warranted, as well as highlighting the lessons learned from historical investigation going forward.

**Abstract:**

Glioblastoma remains one of the deadliest and treatment-refractory human malignancies in large part due to its diffusely infiltrative nature, molecular heterogeneity, and capacity for immune escape. The Janus kinase/signal transducer and activator of transcription (JAK/STAT) signaling pathway contributes substantively to a wide variety of protumorigenic functions, including proliferation, anti-apoptosis, angiogenesis, stem cell maintenance, and immune suppression. We review the current state of knowledge regarding the biological role of JAK/STAT signaling in glioblastoma, therapeutic strategies, and future directions for the field.

## 1. Introduction

Despite decades of intense study, the prognosis for patients with glioblastoma (GBM) remains near universally poor, with inevitable therapeutic resistance and subsequent recurrence despite multimodality therapies. As advances in other solid and liquid cancers continue to be made, there remains an urgent unmet need for GBM therapeutics. Treatment resistance arises from a wide variety of mechanisms, including the blood–brain barrier (BBB), inter- and intra-tumoral heterogeneity, and a profoundly immunosuppressive tumor microenvironment (TME). As the mechanisms underlying these barriers have been and continue to be elucidated, a number of crucial oncogenic signaling pathways have been discovered that contribute redundantly in promoting tumorigenesis, disease recurrence, and confounding therapeutic strategies. Increasing evidence demonstrates the importance of Janus kinase (JAK)/signal transducer and activator of transcription (STAT) signaling as a pivotal molecular hub active in critical microenvironmental cellular populations, such as glioma cells, reactive astrocytes, and stromal and immune cells, that drives not only aggressive growth, invasion, treatment resistance, and cancer cell stemness but also tumor-mediated immunosuppression. Herein, we review the present knowledge to date of JAK/STAT signaling in promoting these activities, the historical approaches taken to target this pathway, and the future directions.

## 2. Physiologic JAK/STAT Signaling

**Figure 1:**
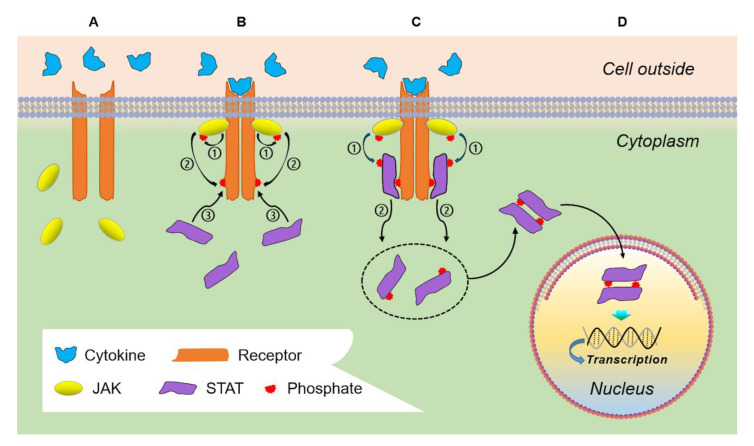
Physiologic JAK-STAT signaling. The classical JAK/STAT signaling pathway begins with (**A**) recognition and binding of an extracellular cytokine by its respective receptor leading to (**B**) receptor dimerization, docking of JAK protein, JAK autophosphorylation, and phosphorylation of the receptor’s cytoplasmic tail. STAT proteins are then able to (**C**) bind via their SH2 domains to these activated receptors, undergoing the critical activating step of tyrosine phosphorylation that allows them to dimerize with other STAT proteins and (**D**) translocate to the nucleus to effect transcription of target genes. Abbreviations: JAK—Janus kinase; STAT—signal transducer and activator of transcription.

The STAT family of transcription factors is comprised of seven proteins—STAT1, STAT2, STAT3, STAT4, STAT-5a, STAT-5b, and STAT6—which reside in the cytoplasm and are activated by phosphorylation as a downstream consequence of a number of signaling pathways, including cytokines, growth factors, or non-receptor tyrosine kinases [1]. In the classical JAK-mediated pathway, cytokine binding of its cognate receptor leads to receptor dimerization followed by docking of JAK and consequent phosphorylation of the receptor’s cytoplasmic tail. STAT proteins are then recruited via their SH2 domains to the activated receptor where tyrosine phosphorylation occurs, STAT hetero- or homodimerization ensues, and activated STAT then undergoes translocation to the nucleus to bind DNA elements such as promoters or enhancers to both directly and indirectly regulate transcription of associated genes (Figure 1) [2]. Although tyrosine phosphorylation of STAT is the most important activating step, STAT can be phosphorylated on serine residues to modulate their activity.

The negative regulation of STAT signaling is mediated through a number of mechanisms acting both upstream and downstream of STAT activation, such as the suppressors of cytokine signaling (SOCS) proteins that inhibit JAK activity via binding to their SH2 domains [3]. The protein inhibitors of activated STAT (PIASs) proteins are another group of proteins that can bind activated STAT and prevent DNA binding, thereby inhibiting downstream transcriptional programs [4,5,6]. Protein tyrosine phosphatases (PTPs), such as Src homology region 2-containing protein tyrosine phosphatase 2 (SHP2), inactivate STAT molecules via dephosphorylation [7,8].

Of the aforementioned seven STAT family members, pathogenic activation of STAT1, STAT3, and STAT5 have been studied in malignant glioma and are the main focus of this review.

## 3. Biological Principles in JAK/STAT Signaling in Glioblastoma Cells

### 3.1. STAT1 and STAT5 in GBM

The role of STAT1 in malignant glioma is evolving. Historically, STAT1 was believed to play a tumor suppressor role; however, more recent studies support a more nuanced view [9,10,11]. For example, exogenous overexpression of STAT1 was initially described to decrease proliferation and migration and increase apoptosis in glioma cells [12]. STAT1 signaling is also a negative regulator of HIF-1α-mediated *CXCR4* and *VEGF* gene expression [9,13]. In direct contrast, a more recent study showed that IL-8 secreted by glioblastoma and other microenvironmental immune cells promotes glioma migration, invasion, and mesenchymal transition via the STAT1/HIF-1α/Snail cascade [14]. Consistently, investigators have found upregulated expression of STAT1 signaling genes to be associated with poor prognosis in pathways involving *N-Myc* or IGFBP-3 [11,14,15].

The complexity of the role of STAT1 in the GBM TME is further confounded by the therapeutic context. For example, STAT1 signaling is essential to induce the effects of oncolytic virotherapy by prompting proinflammatory and apoptotic effects through interferon [16]. Interestingly, STAT1 is expressed in the cytoplasm of reactive astrocytes at the leading or invasive edge of most GBMs as well as in microglia, although the mechanistic implications of this are unclear since it is not the activated phosphorylated form or in a transcriptionally active nuclear location [17]. On balance, it appears to be that the net effect of STAT1 signaling in glioblastoma is context dependent, and this axis may therefore be difficult to therapeutically modulate in a predictable fashion.

STAT5 signaling has been linked to tumorigenesis in GBM. STAT5 is mainly concentrated at the tumor leading edge and plays a role in proliferation and invasion [18,19]. Indeed, knockdown of STAT5 leads to impaired migration in epidermal growth factor variant III (EGFRvIII)-bearing GBM, likely as a result of decreased MMP-1 [20,21]. Granulocyte macrophage colony-stimulating factor (GM-CSF) secreted by glioma cells also activates STAT5 signaling in myeloid derived suppressor cells (MDSCs) to induce Bcl2a1 expression and downregulate *IRF8* transcription, thereby inhibiting apoptosis and promoting proliferation, respectively [22,23]. Overall, STAT5 appears to be a potential therapeutic target in GBM, although a dearth of confirmatory in vivo data currently precludes this application. 

### 3.2. STAT3 Dysregulation in GBM

**Figure 2:**
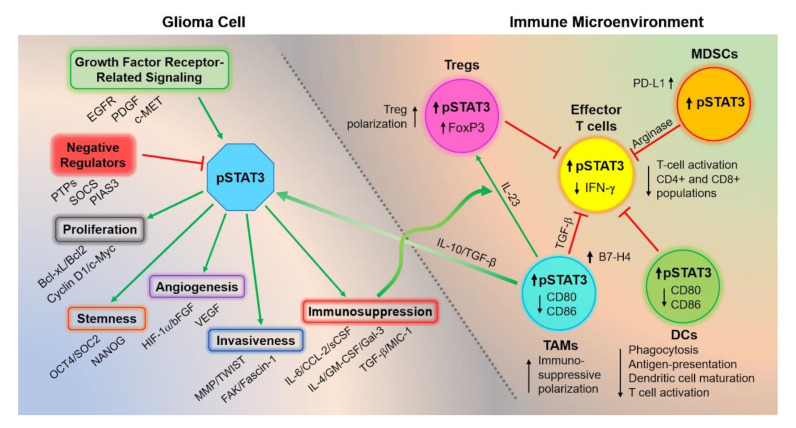
STAT3 pathway activation represents a focal point of tumorigenesis and immune escape. Aberrant STAT3 activation occurs as a result of several potential upstream and downstream regulatory events including growth factor receptor signaling (e.g., epidermal growth factor (EGFR), platelet-derived growth factor (PDGF), and c-MET)), inhibition of negative regulators of STAT3 (e.g., protein tyrosine phosphatases (PTPs), suppressors of cytokine signaling (SOCS), and protein inhibitor of activated STAT 3 (PIAS3)), and microenvironmental cytokine crosstalk between immune and glioma cells. STAT3 activation transcriptionally upregulates key genes involved in proliferation, stem cell self-renewal, angiogenesis, invasiveness, and formation of the immune microenvironment. The balance of microenvironmental cytokines favors the infiltration of immunosuppressive immune cell populations, including myeloid derived suppressor cells (MDSCs), tumor-associated macrophages/microglia (TAMs), and Tregs. These cytokines activate STAT3 signaling within immune cell populations to increase immunosuppressive macrophage polarization, decreased antigen presentation, and decreased T cell activation.

Of all the STAT family members in GBM, STAT3 is the most well-characterized in its breadth of oncogenic activity and immune suppressive role. As gain-of-function STAT3 mutations have not been described in GBM, aberrant STAT3 signaling in malignant glioma occurs predominantly as a result of dysregulated upstream events that ultimately promote proliferation; neovascularization; resistance to apoptosis; and immune escape through downstream targets, such as Bcl-xL, Bcl2l1, Bcl-2, cyclin D1, and c-Myc (Figure 2) [24,25]. Constitutive activation of EGFR signaling—for instance, via EGFR amplification, which occurs in 60% of GBMs—leads to dysregulated STAT3 signaling in part via inhibition of nuclear phosphatases by TRIM59 and constitutive nuclear translocation of STAT3 by RanBP6 [26,27,28,29,30,31]. Basic fibroblast growth factor (bFGF), PDGF, c-MET, and PKCε can also activate STAT3 signaling [32,33]. Elevated expression of IL-6 within the TME by a number of cellular populations, including reactive astrocytes, also leads to increased STAT3 signaling through JAK signaling [34,35].

A lack of negative STAT3 regulation also contributes to its constitutive activation in gliomas. Indeed, epigenetic silencing of the *PTPRD* gene encoding the receptor protein tyrosine phosphatase delta—an inactivating dephosphorylator of STAT3—is commonly found in GBM as a consequence of CpG hypermethylation on chromosome 9p [36]. Additionally, *SOCS1* and *SOCS3* promoter hypermethylation–which inactivates these genes—occurs in 24% and 35% of GBM, respectively, and is associated with poor prognosis [37]. SOCS3 knockdown results in an increase in EGFR-related signaling pathways [37,38,39]. Recently characterized, the non-receptor tyrosine kinase bone marrow and X-linked (BMX) is also an inhibitor of SOCS3 and is highly upregulated in nearly 90% of patient-derived glioma stem cells (GSCs) [40]. Finally, overexpression of TGF-β-related protein Smad6 in nearly 90% of GBM tissues promotes the ubiquitination and subsequent degradation of the STAT3 inhibitor PIAS3 [41,42,43]. As a consequence of these aforementioned mechanisms, the reported frequency of phospho-Tyr-STAT3 positivity in human glioma samples ranges up to 60% and has been significantly correlated with histologic grade, EGFRvIII positivity, aggressive behavior, and poor prognosis [44,45,46,47,48,49]. Recent work has affirmed the negative prognostic significance of upregulation of JAK/STAT gene targets—e.g., cytokines, cytokine receptors, and JAKs—in the classical subtype of GBM [50]. 

Adding a layer of complexity to the role of STAT3 in gliomagenesis is that the role of STAT3 may be to a certain degree genetically determined. In one notable in vitro study, de la Iglesia et al. demonstrated that in GBMs harboring inactivating mutations of *PTEN*, consequent AKT activation led to downregulation of cytokine receptor leukemia inhibitory factor receptor-β and inhibition of STAT3 signaling, thereby leading to IL-8-induced proliferation and invasiveness [51]. A companion study by de la Iglesia et al. further found that in EGFRvIII-positive GBMs, STAT3 complexed with EGFRvIII in the nucleus to activate its tumorigenic activity [52]. In other words, in *PTEN*-deficient GBM (found in ~35% of GBM), STAT3 may be tumor suppressive rather than oncogenic [53]. These findings may have significant implications for the clinical study of STAT3 inhibition in GBM. However, because of the known complexity of STAT3 dysregulation, and because an association between *PTEN* status and response to STAT3 inhibition has not been shown, it would be premature to exclude such a significant proportion of patients with GBM from a clinical trial of a STAT3 inhibitor. *PTEN* status will likely need to be considered in the correlative analysis of outcome in any clinical trial involving STAT3 inhibition in GBM. In summary, the net balance favors aberrant STAT3 pathway activation as primarily tumorigenic in GBM.

### 3.3. Consequences of Dysregulated STAT3 Signaling in GBM

**Figure 3:**
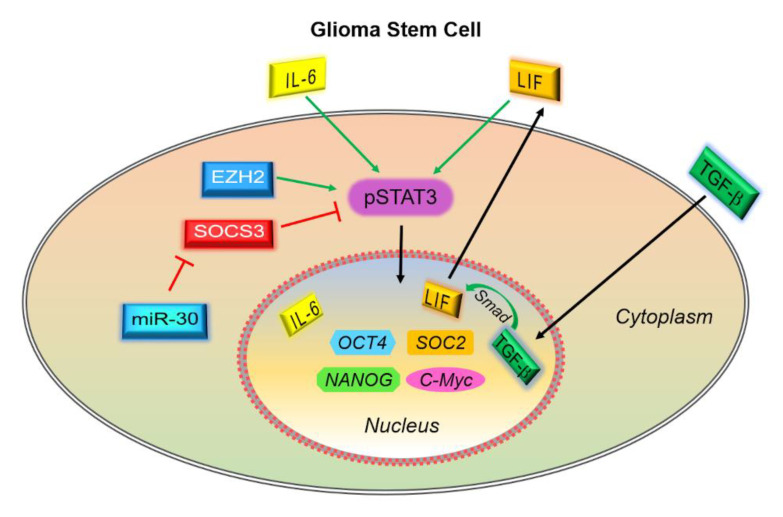
STAT3 activation in glioma stem cells. A number of dysregulated extra- and intra-cellular signaling pathways lead to constitutive STAT3 activation in glioma stem cells, which are important progenitor cell populations that are capable of seeding recurrent disease. Chief among these is aberrant cytokine-related JAK/STAT signaling, which includes both autocrine (e.g., IL-6 and leukemia inhibitory factor (LIF)) and paracrine (e.g., IL-6, transforming growth factor (TGF)-β among others) factors. Intracellular regulators include miR-30 (which binds and inhibits the STAT3-inhibitory protein SOCS3) and enhancer of zeste homolog 2 (EZH2), which methylates STAT3 to promote its constitutive activation. The net effect is to promote self-renewal, prevent apoptosis and differentiation.

STAT3 activation underlies a host of tumorigenic cellular pathways, the foremost of which is in transcriptionally regulating glioma stem cells (GSCs)—a critically treatment-resistant population of cells that give rise to recurrent disease [54,55]. Indeed, activated STAT3 has been associated with Notch signaling in GBMs that is involved in regulating stem cell genes such as *OCT4*, *SOC2,* and *NANOG* [56,57,58]. Aberrant cytokine signaling also promotes GSC self-renewal. One such cytokine is transforming growth factor (TGF)-β, which is highly overexpressed in glioblastoma where it induces expression of the cytokine leukemia inhibitory factor (LIF) to consequently activate JAK/STAT3 to prevent differentiation of the GSCs [59]. Another mediator is IL-6, which is secreted by a multitude of microenvironmental cells that is critical for GSC survival [60,61]. miR-30 is another factor highly expressed in GSCs that binds and inhibits SOCS3 to further promote STAT3 activation [62]. STAT3 also promotes the transcription of miR-182-5p, which binds and inhibits the tumor suppressor protocadherin-8 [63,64]. Finally, STAT3 and NF-κB are preferentially bound and methylated by the enhancer of zeste homolog 2 (EZH2) in GSCs, enhancing their activation and self-renewal [65,66]. These are summarized in Figure 3.

The multi-faceted tumorigenic roles of STAT3 in human glioma cells has been well established by both molecular and pharmacologic inhibition. Targeted inhibition of STAT3 in GSCs not only triggers apoptosis but also leads to decreased proliferation, multipotency, and neurosphere formation [67,68,69]. RNAi-mediated knockdown of STAT3 in human U251 glioma cells led to increased apoptosis via the suppression of transcriptional downstream targets of STAT3, such as *Bcl-xL*, *Mcl-1,* and *survivin* [70,71,72]. WP1066, a small-molecule STAT3 inhibitor, similarly induced apoptosis in U87-MG cells and murine xenografts [73]. 

Angiogenesis is another hallmark of cancer that has been directly linked to STAT3. In histopathologic examination, phosphorylated STAT3 preferentially localizes to tumor endothelial cells along with vascular endothelial growth factor receptor-2 (VEGFR-2), suggesting an autocrine feed-forward loop promoting the hallmark neovascularization seen in GBM [74]. Indeed, tissue hypoxia dose-dependently increases STAT3 phosphorylation and consequent angiogenesis in human GBM cell lines by stabilizing HIF-1α to enable its transcription of *VEGF* [75]. GBM also responds to hypoxia by STAT3-mediated upregulation of key proteins involved in motility and invasiveness, such as the matrix metalloproteinases (MMPs-2, -3 and -9), focal adhesion kinase, fascin-1, and TWIST [76,77,78,79,80]. Additionally, activated STAT3 promotes the epithelial–mesenchymal transition (EMT) and invasive behavior [81,82].

### 3.4. Consequences of Dysregulated STAT3 Signaling on the Immune Microenvironment

With the surging enthusiasm for immunotherapy for other solid malignancies, immune therapeutic targeting of STAT3 is a compelling strategy for modulating tumor-mediated immune suppression, especially for a cancer like GBM that has in general not been responsive to immune checkpoint inhibitor monotherapies [83,84]. Indeed, GBMs have been shown to express a number of tumor-specific antigens—e.g., EGFRvIII, IL13Rα2, and HER2—that may be recognized by the host immune system [85,86,87,88], yet there is compelling evidence that glioma-mediated immune suppression prevents immune recognition and effector responses. For example, GBM is notable for a paucity of T cells and an enrichment of myeloid cells such macrophages and microglia, which may be even more evident in the transcriptionally defined GBM mesenchymal and classical subtypes [89,90]. As will be discussed, a substantial body of literature points to the role of aberrant STAT3 signaling in mediating dysfunction of both the innate and adaptive components of the immune system in GBM [91,92].

#### 3.4.1. STAT3 Activation Generates an Immunosuppressive Cytokine Milieu

In GBM, interactions between tumor cells, reactive astrocytes, and microglia lead to high levels of IL-10 and TGF-β, which promotes a positive feedback loop of STAT3 signaling [93,94]. STAT3-driven HIF-1α transcription also leads to the secretion of immunosuppressive cytokines, such as galectin-3, CCL-2, and CSF. These events, combined with additional STAT3-driven elaboration of such factors as IL-4, IL-6, and GM-CSF, further promotes immunosuppressive crosstalk [55,95,96,97,98,99,100]. Anti-sense oligonucleotide-mediated STAT3 blockade in murine models of melanoma and breast and colon cancer show upregulation of proinflammatory cytokines, such as CXCL10, RANTES, TNF-α, and IFN-β [101]. 

#### 3.4.2. STAT3 Activation Impairs the Innate Immune System

Preferential activation of the STAT3 pathway by the permissive cytokine milieu has profound effects on the innate immune system components of the TME, which are largely mediated by tumor-associated macrophages/microglia (TAMs), myeloid-derived suppressor cells (MDSCs), and other cells [102]. Natural killer (NK) cells in the setting of STAT3 activation, for instance, have impaired cytotoxicity [92]. STAT3 activation in GBM-resident TAMs, which comprise the largest population of infiltrative cells, leads to polarization toward an immunosuppressive phenotype that secretes IL-10 and TGFβ1 and are impaired in their ability to mediate phagocytosis [103,104,105,106,107,108]. These same TAMs also lack co-stimulatory molecules such as CD80 necessary for T cell activation and secrete IL-23, which induces Tregs to adopt a more immunosuppressive phenotype [109,110]. 

STAT3 activation also inhibits maturation of dendritic cells and their ability to express key molecules necessary for effective antigen presentation (e.g., MHC class II) and T cell activation (e.g., IL-12) [92,111]. Similar to its effect in TAMs, STAT3 also inhibits the expression of co-stimulatory molecules CD80 and CD86 on dendritic cells that are necessary for induction of T cell activation and proliferation.

Various cytokines—but particularly IL-6—are associated with the infiltration of MDSCs in the TME, which are positively correlated with glioma grade and have been shown to exert immune suppressive effects against T and NK cells through expression of enzymes such as arginase that trigger T cell arrest and apoptosis [112,113,114,115,116,117]. MDSCs also express IFN-α, which signals through interferon receptor type 1 (IFNAR1) to activate JAK1/STAT1 signaling, thereby upregulating expression of co-inhibitory molecule programmed death ligand-1 (PD-L1) [114]. STAT3 inhibition not only promotes the maturation of tumor-infiltrating dendritic cells to express the costimulatory molecules, such as CD80 and CD86, necessary for T cell activation, but also induces a shift in tumor immune cell composition toward less MDSCs and immunosuppressive macrophages/microglia [118,119,120,121].

#### 3.4.3. STAT3 Activity Impairs the Adaptive Immune System

Constitutive STAT3 activation also has significant effects on the adaptive immune system. STAT3 activation alters the immune cell composition of the microenvironment, promoting decreased infiltration of effector CD4+ and CD8+ T cells and an increased proportion of Tregs [121]. Activation of STAT3 in CD8+ T cells reduces their secretion of IFN-γ, which further inhibits T cell activation [122]. STAT3 cooperates with transcription factor Forkhead box P3 (FOXP3) to promote the differentiation of immunosuppressive Tregs [123,124,125,126,127]. Indirectly through innate immunity, TAMs inhibit T cell proliferation via secretion of TGF-β [105]. STAT3 has also been shown to play an important role in regulating PD-L1 expression by antigen-presenting dendritic cells and in upregulating the expression of T cell coinhibitory molecule B7-H4 on GSCs and TAMs [95]. 

In a pivotal early study, Kortylewski et al. demonstrated that hematopoietic cell-specific inhibition of STAT3 in tumor-bearing mice led to significantly enhanced functional activity of T, NK, and dendritic cells, resulting in antitumor immunity and growth inhibition [92]. Hussain et al. similarly demonstrated that STAT3 inhibition of the immune cells from glioblastoma patients promotes not only expression of co-stimulatory molecules on microenvironmental TAMs and proinflammatory cytokines but also the expansion of effector T cells over immunosuppressive Tregs [122].

In summary, STAT3 signaling constitutes a crucial hub through which the tumor microenvironment is shaped and driven toward immunosuppression. 

## 4. STAT3-Mediated Resistance to Therapeutic Modalities

At present, the standard-of-care for patients with GBM consists of maximum safe resection followed by the “Stupp regimen”: alkylating chemotherapy temozolomide (TMZ) combined with radiation, followed by adjuvant TMZ. TMZ mediates its cytotoxic effects via the formation of O6-methylguanine adducts on DNA, leading to replication-associated double-stranded DNA breaks and apoptosis [128,129]. A number of studies have shown that STAT3 signaling is involved in chemoresistance. Kohsaka et al. demonstrated that STAT3 inhibits the degradation of the enzyme O(6)methyl-guanine DNA methyltransferase (MGMT) [130], whose expression is associated with TMZ resistance [131]. Additionally, findings from Wang et al. [132] and Lee et al. [133] indicate that inhibition of STAT3 sensitizes glioma cells to TMZ. Another study by Li et al. linked a reduced level of the STAT3 inhibitory miR-519a—which normally mediates proapoptotic and autophagic responses to chemotherapy—to TMZ resistance in GBM cells [134]. They further showed that STAT3 inhibition strongly decreased the IC_50_ of TMZ and increased TMZ-induced apoptosis while upregulating Bcl-2 expression and downregulating Bax expression. Finally, they showed that STAT3 inhibition leads to increased TMZ-induced G0-G1 arrest and decreased cyclin D1 expression compared to TMZ alone. It should be noted that these findings are somewhat contradicted by recent work from Heynckes et al. [135], which found that recurrent GBM tissue from Stupp regimen-treated patients demonstrated decreased phospho-STAT3 expression compared to their original tumors. Furthermore, the authors found that treatment of two IFNγ-stimulated patient-derived GBM cell lines with TMZ led to decreased JAK/STAT pathway genes including *STAT1* and *STAT3*, although this was not seen in the commercial LN229 GBM cell line. The generalizability of the authors’ primary conclusion that TMZ treatment leads to inhibition of JAK/STAT signaling in recurrent GBMs is uncertain. Notably, this study had methodological limitations, including small sample sizes (three cell lines and fifteen tissue samples) and—most critically—the absence of microenvironmental JAK/STAT expression analysis. Nevertheless, the authors’ findings suggest that the role of aberrant STAT3 signaling in underlying chemoresistance is nuanced and further point to the fact that the optimal role and timing of STAT3 inhibition in combination with conventional therapeutic strategies remains to be determined.

There is also evidence that STAT3 is involved in mediating resistance to radiotherapy, which induces cell death largely via single- and double-stranded DNA breaks and oxidative damage [136]. Rath et al. found that cytokine crosstalk between astrocytes and GSCs in vitro led to transcriptional upregulation of STAT3 target genes and radioresistance in the latter [137]. Radiation combined with pharmacological inhibition of STAT3 in the corresponding orthotopic murine xenografts led to decreased tumor size and prolonged survival, suggesting that STAT3 contributes to the baseline radioresistance of GSCs. More recently, Yu et al. elucidated that this may involve the protein regulator of chromosome condensation 2 (RCC2), which signals through STAT3 to activate transcription of DNA methyltransferase 1 to lead to silencing of tumor suppressor genes [138]. Radiation induces the secretion of exosomes by glioma cells containing proteins involved in numerous crucial signaling pathways including JAK/STAT, as well as proteins such as ribophorin II, which signal through STAT3 to promote anti-apoptosis via Mcl1 [139,140]. Studies of GBM cells with acquired radioresistance are notable for decreased SOCS3 and increased Forkhead box protein M1 (FOXM1), the latter of which interacts with STAT3 to increase transcription of DNA repair genes such as *MRE11* and *RAD51* [141,142]. Finally, irradiated GBM cells have been shown to acquire increased invasive, migratory, and mesenchymal properties via the upregulation of intercellular adhesion molecule (ICAM-1) through activated STAT3/NF-κB and Slug signaling [143,144]. In summary, STAT3 signaling appears to be involved in promoting intrinsic as well as acquired chemo- and radio-resistance. 

There is substantial literature that supports the role of STAT3 activation in mediating resistance to targeted therapy. The reliance on several dysregulated—and often redundant—signaling pathways, including EGFR, PIK3CA, PDGF, and NF-κB, has been well-characterized in glioblastoma, and cross-talk between these have likely contributed in large part to the historical failures of targeted therapy [53,145,146]. Indeed, compensatory STAT3 upregulation has been demonstrated in numerous studies of other receptor tyrosine kinases (e.g., EGFR, MEK, HER2) in other solid tumors [147,148,149]. Treatment of GBM with anti-VEGF monoclonal antibody bevacizumab for instance—currently the only FDA-approved targeted treatment for glioblastoma and one that has not been shown to have survival benefit—eventually induces STAT3 activation likely via the hypoxia response, leading to the expression of stemness and invasive markers such as nestin and ICAM-1, respectively [150]. The combination of STAT3 and VEGF inhibition with AZD1480 and cediranib, respectively, led to significantly decreased angiogenesis and tumor volume in a murine model [150,151]. In a more recent study of acquired MET inhibitor resistance in GBM, Cruickshanks et al. found compensatory upregulation of a number of other signaling pathways, including MAPK, PI3K, and STAT [152]. Co-inhibition of STAT3 and MET restored sensitivity to apoptosis.

### Rationale for Combination Therapy

From the preceding discussion, it is clear that the compensatory mechanisms of resistance to conventional, targeted, and immunotherapeutic strategies against GBM will likely thwart therapies based on a single vulnerability [153]. Consequently, one of the major tasks in neuro-oncology is leveraging continually evolving molecular knowledge to design rational therapeutic combinations. One recently published study of STAT3 inhibition and radiation in a syngeneic immune-competent glioma mouse model found that the combination led to immunologic reprogramming of the TME, with increased dendritic cell–T cell interaction and antigen presentation. This was associated, moreover, with significantly improved animal survival, indicating that a fully functional immune response was required for mediating the therapeutic effects of STAT3 inhibition [154]. Clinical investigators have also recognized that STAT3 inhibition as monotherapy is unlikely to demonstrate an improvement in survival for the vast majority of GBM patients, and, as such, the results of trials investigating STAT3 inhibition with conventional radiation (NCT03514069) and chemotherapy (NCT02315534) are eagerly awaited. 

With regard to its well-characterized immunomodulatory effects, combining STAT3 inhibition with other immuno-oncologic strategies also represents an exciting prospect. For example, one preclinical study has shown improved effector functions of adoptively transferred CD8+ T cells in combination with STAT3 inhibition in myeloid cells [155]. Another study demonstrated that administration of a STAT3-targeting miR-124 in combination with a T cell co-stimulatory aptamer was able to increase CD4+ and CD8+ T cells and decrease macrophage trafficking into the TME in a genetically engineered murine glioma model [156]. Subsequent immunotherapeutic efforts focusing on further optimizing tumor-specific cytotoxicity and immune microenvironmental modulation are clearly warranted. The combination of STAT3 inhibition with other immunotherapies, such as adoptive cell transfer, tumor vaccination, oncolytic virotherapy, and immune checkpoint inhibition, are rational. Trials investigating the safety and efficacy of STAT3 and PD-1 blockade (e.g., NCT02851004, NCT02467361, NCT02983578, and NCT03421353) in various solid malignancies are underway, and there is hope that insights gained from these may inform trial design for GBM.

## 5. JAK/STAT Axis-Targeting Therapies

The aforementioned convergence of multiple oncogenic and immunosuppressive cellular pathways on the JAK/STAT signaling axis makes it an attractive molecular target. Indeed, Stechishin et al. found that targeted JAK2/STAT3 inhibition in orthotopic GBM xenografts regardless of molecular strategy led to increased cytotoxicity of GSCs regardless of MGMT promoter methylation status [157]. To date, a vast number of agents ranging from antisense oligonucleotides and repurposed drugs, JAK1/2 to direct STAT3 inhibitors have been the subject of investigation in numerous cancers. As the BBB presents a unique anatomic barrier to drug delivery in contrast to other malignancies, identifying high-potency, specific, BBB-penetrant molecules of favorable bioavailability is the foremost priority. A discussion of those studied in malignant glioma follows. 

Targeting aberrant upstream IL-6/IL-6R signaling is one potential avenue of JAK/STAT blockade. Treatment with IL-6 pathway blockade via its receptor (IL-6R, tocilizumab) or binding soluble IL-6 (siltuximab) has been shown to inhibit glioma growth in vitro and reduce the expression of coinhibitory molecules such as PD-L1 on infiltrative myeloid cells [158,159]. A number of clinical trials investigating the efficacy of these agents in other solid malignancies are underway (e.g., NCT03135171, NCT04258150 NCT04524871, NCT03424005, and NCT04191421), although it should be noted that as far as potential therapies for GBM, these large monoclonal antibodies have not been found to attain therapeutic concentrations in the CSF after systemic administration [160]. 

A number of repurposed pharmacologic agents have been found to have STAT3 inhibitory activity; however, off-target effects due to lack of specificity and questionable CNS penetrance have limited their utility. Atovaquone is an anti-malarial drug FDA-approved for pneumocystis pneumonia that was found to have STAT3 inhibitory effects; notably, it appears to be poorly bioavailable in the CNS [161,162]. Arsenic trioxide (ATO) was shown to reduce STAT3 activation via JAK inhibition and induce apoptosis and stemness of GSCs. Despite encouraging safety data, a phase II trial combining ATO with radiation and temozolomide for newly diagnosed malignant glioma did not demonstrate a survival benefit [163,164,165]. Sorafenib, a multi-kinase (Raf, VEGFR2, and PDGFR-β) inhibitor with STAT3 inhibitory activity FDA approved for other solid malignancies, was shown to inhibit the proliferation of GBM in vitro, likely through its effects on AKT and MAPK [166,167]. Subsequent clinical trials combining sorafenib with mTOR inhibition, temozolomide, or radiation for patients with newly diagnosed or recurrent GBM did not demonstrate a survival benefit [168,169,170].

### 5.1. Nutraceutical Inhibition of STAT3

A staggering number of natural compounds have been found to have STAT3 inhibitory activity in vitro, but the clinical utility of these in their native forms for GBM is largely hindered by unfavorable pharmacokinetic properties including low potency, unacceptable toxicity, rapid metabolism, and/or poor BBB penetrance. Plant/fruit-derived resveratrol, for instance, is capable of inhibiting proliferation and invasion of glioma cells in a STAT3-dependent fashion in vitro and when administered intrathecally in vivo. Sadly, it is rapidly metabolized when given systemically [171,172]. To circumvent this, Jhaveri et. al., encapsulated resveratrol within transferrin-bearing liposomes targeting GBM cells [173]. This strategy was insufficient in that no overall significant growth inhibition after intravenous administration was seen. Earlier efforts with liposomal modification of ursolic acid met with similar lack of anti-proliferative and anti-angiogenic activity [174]. What appears to be increasingly clear is that the chief utility of natural STAT3-inhibiting compounds lies in serving as the structural basis for synthesizing novel and more effective small molecules. KYZ3, for instance, a synthetic derivative of quinoid diterpene cryptotanshinone found in dried roots, demonstrated cytotoxic activity in a murine model of triple-negative breast cancer [175]. The various challenges associated with a selected list of natural compounds are summarized in Table 1.

### 5.2. Pharmaceutical Inhibition of STAT3: JAK Inhibitors

JAK inhibition, although historically employed in the treatment of myeloproliferative disorders, represents another avenue of STAT3 pathway targeting in malignant glioma. As summarized in Table 2, a number of agents have encouraging preclinical evidence of antitumoral efficacy in glioma cells or stem cells, although confirmatory in vivo studies are pending (e.g., G6 and SAR317461) [211,212]. One agent is JSI-124, a JAK2 inhibitor with NF-κB pathway activating capability based on the structure of cucurbitacin, which has been shown to inhibit proliferation of glioblastoma in vitro and also promote the maturation and T-cell-activating capability of dendritic cells isolated from the spleens of tumor-bearing mice, leading to improved cytotoxicity and growth inhibition with subsequent dendritic cell vaccination [209,213,214,215]. To date, only one JAK inhibitor of potential benefit for GBM patients—AZD1480—has advanced to a phase I clinical trial, where unusual dose-limiting neuropsychiatric toxicities halted further development [216,217]. The most recent JAK inhibitors of translational relevance are pacritinib and ruxolitinib. Both are orally administered, BBB-penetrant, highly potent inhibitors of either JAK1/JAK2 (ruxolitinib) or JAK2 (pacritinib), with demonstrated GSC-specific cytotoxicity and chemosensitizing properties [218]. Interestingly, pacritinib was also shown to decrease the amount of miR-21-enriched exosomes released from tumor-associated macrophages, reducing an exogenous source of STAT3 upregulation in glioma cells [94]. Ruxolitinib not only decreased the invasiveness of GBM cells but also was found to be able to inhibit the compensatory JAK1/STAT1 signaling that limits the efficacy of oncolytic virotherapy [219,220,221]. Ruxolitinib is currently being evaluated in a phase I trial for patients with newly diagnosed MGMT-unmethylated malignant glioma in combination with radiation and temozolomide (NCT03514069). 

### 5.3. Pharmaceutical Inhibition of STAT3: Peptide and Non-Peptide STAT3 Mimetics

Peptidomimetics such as PY*LKTK were among the earliest in vitro STAT3 inhibiting strategies, designed to bind the SH2 domain and thereby prevent dimerization and DNA binding [222]. Concerns about in vivo stability, immunogenicity, and low potency led to the design of non-peptide mimetics, such as LLL12 and STX-0119, which have been found to induce apoptosis in GBM cell lines or TMZ-resistant xenografts via inhibition of downstream STAT3 targets, such as survivin Bcl-2 and Bcl-xL [223,224,225,226]. In spite of these, moderate potency (e.g., IC_50_ 15–44 µM for STX-0119), poor bioavailability, and/or unclear BBB penetrance limit the utility of these agents at this time.

### 5.4. Pharmaceutical Inhibition of STAT3: Oligonucleotide-Based Strategies

Oligonucleotides represent another strategy of direct STAT3 modulation. In a proof-of-concept experiment, Gu et al. designed a decoy oligodeoxynucleotide comprised of a STAT3-specific DNA *cis*-element and transfected it into human GBM cells U251 and A172, finding specific blockade of STAT3 activation with subsequent cell-cycle arrest and apoptosis mediated by decreases in mRNA levels of c-Myc, cyclin D1, and Bcl-xL [71]. Kortylewski et al. conjugated a STAT3-specific small interfering (siRNA) to a Toll-like receptor agonist CpG and found it was able to be internalized by tumor-associated macrophages and dendritic cells to mediate STAT3 inhibition [227]. MicroRNA (miRNA)-based strategies have also shown promise. Having initially found STAT3-inhibitory miR-124 expression to be significantly downregulated in gliomas compared to normal brain, Wei et al. demonstrated that exogenously administered miR-124 induced secretion of proinflammatory cytokines from GSCs, promoted CD8+ T cell effector with decreased Treg function, and led to immune-mediated growth inhibition in a murine model of glioma [228]. 

As oligonucleotides are susceptible to degradation by circulating nucleases, subsequent groups have developed various modifications to optimize stability and drug delivery. Yaghi et al. encapsulated miR-124 duplexes within lipid nanoparticles and demonstrated efficient uptake by immune cells with subsequent reduction in activated STAT3 and increased survival in a murine model of glioma [229]. Linder et al. complexed anti-STAT3 siRNA with a polyethylenimine (PEI) and phospholipid 1,2-dipalmitoyl-sn-glycero-3-phosphocholine liposomal conjugate and found it to mediate STAT3 inhibition in glioma cells, although in vivo no significant growth inhibition was observed, and tumoral STAT3 inhibition was quite heterogeneous upon histopathologic examination [230]. PEI-siRNA directed against survivin has also shown some efficacy, albeit in subcutaneously implanted gliomas in mice [231]. 

Overall, the major challenges for nanoparticle-based delivery of oligonucleotides include ensuring adequate tissue distribution in tumors with a high degree of vascular heterogeneity and minimizing nonspecific uptake by non-tumor or stromal cells. Further study is certainly warranted to optimize these approaches before clinical application.

### 5.5. Pharmaceutical Inhibition of STAT3: Direct STAT3 Inhibitors

A tremendous number of molecules have been synthesized to inhibit STAT3, usually acting by binding the SH2 domain required for interacting with phosphorylated tyrosine residues on receptors and STAT dimerization. Of these, only a handful have advanced to clinical trials for GBM, for reasons that will be discussed. 

Stattic was one of the earliest SH2-binding STAT3 inhibitors, and was found to exert anti-proliferative and radiosensitizing effects on GSCs, although a lack of specificity and in vivo efficacy data has prevented it from moving forward [232,233,234,235]. AG490 is a caffeic acid derivative able to inhibit STAT3 phosphorylation in vitro to decrease the invasiveness of GBM cells, whose low pharmacologic potency has also hindered further development [73,236]. Promisingly, SH-4-54 is small molecule STAT3/STAT5 inhibitor based on salicylic acid with excellent BBB penetration that was found to potently induce apoptosis in GSCs and inhibit growth of gliomas in a murine xenograft model, although it should be noted that these were subcutaneously implanted tumors [237,238]. Another study by Cui et al. showed that SH-4-54 could induce apoptosis in TMZ-resistant GBM cells by inducing the translocation of mitochondrial STAT3 [239]. This led to the activation of mitochondrial STAT3, negative regulation of mitochondrial-encoded genes, and abnormal oxidative phosphorylation.

Attempts to improve the pharmacokinetic parameters of small molecules such as AG490 led to the development of subsequent compounds such as WP1193 [240] and one of the best-studied novel STAT3 inhibitors, WP1066. WP1066 demonstrated not only favorable BBB penetrance and proapoptotic effects in GBM cell lines but also the important capability of upregulating immune costimulatory molecule expression and the release of proinflammatory cytokines from TAMs. Indeed, this molecule was among the first to document the modulating effects of STAT3 on immune cell composition and effector function in the TME [73,122,241]. It has recently been shown to have radiosensitizing effects in GSCs [232]. It is currently the subject of study in a phase I clinical trial of patients with recurrent glioblastoma (NCT01904123) and pediatric patients with brain tumors (NCT04334863). Napabucasin is another recently characterized orally bioavailable small-molecule STAT3 inhibitor capable of inducing cell cycle arrest, apoptosis, and the reduction of markers of stemness, leading to improved survival in an orthotopic murine glioma model [242,243]. A phase I/II study of napabucasin in combination with TMZ for patients with recurrent glioblastoma has been completed, and the results are eagerly awaited (NCT02315534). The above discussed pharmacologic inhibitors of JAK/STAT signaling are summarized in Table 2.

## 6. STAT3-Related Biomarkers

In view of the tremendous intra- and inter-tumoral heterogeneity of GBM, identifying patients most likely to benefit from targeted STAT3 inhibition is a critically important challenge. There is yet to be a consensus, for instance, on whether expression of phospho-Tyr705 STAT3, phospho-Ser727 STAT3, or both represents maximally abnormal STAT3 pathway activation with its concomitantly poorest prognosis [47,244]. Interestingly, recent work by Tan et al. integrating the TCGA subtypes with STAT3-related gene expression data defined “STAT3-high” and “STAT3-low” gene signatures intended to assist with patient stratification [245]. STAT3-high tumors were enriched for classical/mesenchymal subtyping, IDHwt, and 1p/19q-non-codeleted status, while STAT3-low tumors were enriched for proneural subtypes with IDHmut and 1p/19q-codeleted status. Cells isolated from patients with STAT3-high gene signatures had lower IC_50_s upon treatment with STAT3 inhibitors such as Stattic or WP1066 relative to those with STAT3-low gene signatures. Of note, STAT3-low tumors (i.e., STAT3 inhibition non-responders) upregulated insulin-like growth factor binding protein 2 (IGFBP2) and insulin growth factor-1R (IGF-1R) in response to STAT3 inhibition, and the combination inhibition of IGF-1R and STAT3 in orthotopic xenografts led to re-sensitization to STAT3 inhibition. Interestingly, their functionally determined gene signature outperformed conventional immunohistochemically determined phospho-STAT3 expression in identifying STAT3 inhibition responders, although an important methodological limitation to note is that these were validated in severe combined immunodeficiency (SCID) mice who by definition lack an antitumoral immune response. In addition, it is also notable that the authors did not comment on whether *PTEN* mutation was associated with a “STAT3-low” phenotype, as might be expected based on work by de Iglesia et al. [51]. Future correlative studies based on molecular data of patients being treated with STAT3 inhibitors are warranted to refine the criteria by which patients should be considered for STAT3 inhibition.

## 7. Conclusions

As increasingly sophisticated genomic, transcriptomic, proteomic, and bioinformatic analyses continue to propel the field of oncology into the molecular era, glioblastoma continues to represent a glaringly unmet need. Rational therapeutic strategies need to account not only for anatomic barriers such as the BBB but also the tremendous intra- and intertumoral heterogeneity that characterize this disease. As the preclinical studies have shown, JAK/STAT signaling is tremendously complex, and, while on balance, constitutive activation tends to promote tumor proliferation, angiogenesis, and immune escape, the effects of targeting upstream or downstream effectors are not always predictable. Nevertheless, it is our position that, on balance, there is sufficient evidence of the importance of dysregulated JAK/STAT signaling as an important driver of gliomagenesis and treatment resistance that human study continues to be warranted for this deadly disease. Because it is unlikely that strategies based on one or two key molecular vulnerabilities will be generalizable to the entire patient population, continued efforts to define and validate biomarkers to help stratify patients appropriate for JAK/STAT combination therapy are crucial to advancing our understanding of the disease. 

## Figures and Tables

**Table 1:** Natural compounds with STAT3 inhibitory activity evaluated in glioma.

Natural Compound	Mechanism	Preclinical Evidence of Efficacy in Glioma	Limitations	References
Silibinin	STAT3 inhibition, autophagy, chemosensitization	In vitro	Poor oral bioavailability, low potency	[176,177,178,179,180]
Cryptotanshinone	STAT3 inhibition	In vitro	Poor bioavailability	[181,182]
Alantolactone	STAT3, NF-κB inhibition	In vitro	Poor bioavailability, rapid metabolism, low potency	[183,184]
Shikonin	STAT3, EGFR inhibition	In vitro	Poor bioavailability, low potency	[185,186]
Sulforaphane	JAK2, STAT3, NF-κB inhibition	In vitro	Poor bioavailability, moderate potency	[187,188,189]
Crocetin	STAT3 inhibition (SHP-1 induction)	In vitro	Poor bioavailability, limited BBB penetrance, low potency	[190,191,192]
Cardamonin	STAT3 inhibition	In vitro	Poor bioavailability	[193,194]
Serenoa repens (Saw palmetto)	STAT3 inhibition	In vitro	Poor bioavailability	[195]
Oroxylin A	mTOR, STAT3 inhibition	In vitro	Poor bioavailability, rapid metabolism, low potency	[196,197,198]
Quercetin	IL-6, STAT3 inhibition	In vitro	Poor bioavailability, rapid metabolism, low potency	[199]
Oleanolic acid	STAT3 inhibition, IL-10 inhibition	In vitro	Poor bioavailability, rapid metabolism, low potency	[200,201,202]
Cucurmin	JAK1, JAK2, STAT3 inhibition	In vitro	Poor bioavailability, low potency	[203,204,205]
Ascochlorin	FAK, STAT3 inhibition	In vitro	Poor bioavailability, low potency	[206,207]
Cucurbitacin	JAK, STAT3, PI3K, MAPK inhibition	In vitro	Poor bioavailability, specificity, high toxicity	[208,209]
Resveratrol	STAT3 inhibition	In vitro In vivo	Poor bioavailability, low potency	[171,172,173,210]

**Table 2:** Pharmacologic inhibitors of STAT3 signaling investigated in glioma.

Agent	Mechanism	Evidence of Efficacy in Glioma	Notes	References
G6	JAK2	In vitro	In vivo studies lacking, therapeutic requirement for JAK2 overexpression	[211]
SAR317461	JAK2	In vitro	In vivo studies lacking, compensatory autophagy	[212]
AZD1480	JAK1/JAK2	In vitro In vivo	Unacceptable dose-limiting toxicities	[150,216,217]
JSI-124	JAK2	In vitro In vivo	Anti-proliferative, immune modulatory	[213,214,215]
Pacritinib	JAK2	In vitro In vivo	BBB penetrant, chemosensitizing	[94,218]
* Ruxolitinib	JAK1/JAK2	In vitro In vivo	BBB penetrant, anti-proliferative, radiosensitizing, immune modulatory	[219,220]
PY * LKTK	STAT3	In vitro	In vivo studies lacking, low potency	[222]
LLL12	STAT3	In vitro In vivo	Potent, low solubility/poor bioavailability, unclear BBB penetrance	[223,224]
STX-0119	STAT3	In vitro In vivo	Minimal growth inhibition of GBM in mouse model	[225,226]
AG490	STAT3, JAK2	In vitro	Low potency, in vivo efficacy lacking	[73,236]
Stattic	STAT3, STAT1, STAT2	In vitro	Susceptible to intracellular modification, in vivo efficacy lacking, low specificity	[232,233,234,235]
WP1193	STAT3, JAK2	In vitro	In vivo efficacy data lacking	[240]
SH-4-54	STAT3 STAT5	In vitro In vivo	BBB penetrant, potent, specific, in vivo studies in subcutaneously implanted GBMs	[237,238,239]
* Napabucasin (BBI608)	STAT3	In vitro In vivo	Bioavailable, BBB penetrant	[242,243]
* WP1066	STAT3, JAK2	In vitro In vivo	Bioavailable, BBB penetrant, immune modulatory	[73,122,241]

* Currently being evaluated in clinical trials.

## Data Availability

No new data were created or analyzed in this study. Data sharing is not applicable to this article.
